# Oral Health among Elderly, Impact on Life Quality, Access of Elderly Patients to Oral Health Services and Methods to Improve Oral Health: A Narrative Review

**DOI:** 10.3390/jpm12030372

**Published:** 2022-02-28

**Authors:** Michael Janto, Raluca Iurcov, Cristian Marius Daina, Daniela Carmen Neculoiu, Alina Cristiana Venter, Dana Badau, Adrian Cotovanu, Marcel Negrau, Corina Lacramioara Suteu, Monica Sabau, Lucia Georgeta Daina

**Affiliations:** 1 Faculty of Medicine and Pharmacy, Doctoral School, University of Oradea, 1 December Sq., 410081 Oradea, Romania; michael.janto@t-online.de; 2 Dentistry Department, Faculty of Medicine, University of Oradea, 410073 Oradea, Romania; raluirimie@yahoo.com; 3 Psycho-Neurosciences and Recovery Department, Faculty of Medicine and Pharmacy, University of Oradea, 1 December Sq., 410081 Oradea, Romania; cristi_daina@yahoo.co.uk (C.M.D.); suteucorina@gmail.com (C.L.S.); sabau.monica@yahoo.com (M.S.); lucidaina@gmail.com (L.G.D.); 4 Faculty of Medicine, Transilvania University, 500068 Brasov, Romania; 5 Department of Morphologycal Sciences, Faculty of Medicine and Pharmacy, University of Oradea, 1 December Sq., 410081 Oradea, Romania; 6 Faculty of Sciences and Letters, “George Emil Palade” University of Medicine, Pharmacy, Sciences and Technology, 540142 Targu Mures, Romania; dana.badau@umfst.ro; 7 Interdisciplinary Doctoral School, Transilvania University, 500068 Brasov, Romania; 8 Faculty of Medicine, “George Emil Palade” University of Medicine, Pharmacy, Sciences and Technology, 540142 Targu Mures, Romania; adrian.cotovanu@umfst.ro; 9 Department of Surgical Disciplines, Faculty of Medicine and Pharmacy, University of Oradea, 1 December Sq., 410081 Oradea, Romania; negrau.marcel@gmail.com

**Keywords:** dental health, elderly dental pathology, caries, oral cancer in the elderly, edentulism, access of dental health services, periodontal disease

## Abstract

Dental health is often neglected among the elderly because of the numerous comorbidities in this population, such as cardiovascular diseases. However, dental health influences general health and quality of life by impacting both the general health and the psychological state of the individual. The present review highlights the main dental comorbidities in the elderly population, their impact on the quality of life, the barriers towards access to dental care in the elderly and methods to improve their dental health. Information related to dental care and its importance must be provided both to older individuals and their caregivers in order to detect dental pathology and treat it adequately. Ensuring dental health involves the whole society of elders, caregivers, dental care providers, the public sector, health policymakers, and the private sector.

## 1. Introduction

Health promotion among the elderly is an important preoccupation of healthcare providers and government agencies in order to increase the quality of life of these individuals [1]. Developing diseases in older age is the consequence of both environmental risk factors and genetic factors. It is known that chronic exposure to certain risk factors and the alteration of genetic material can lead to numerous chronic comorbidities [2]. Oral health is often not at the center of attention when discussing health problems among the elderly, a category of population where numerous comorbidities appear, especially cardiovascular or neoplastic pathologies that severely impact the quality of life of this population [3,4].

However, dental pathology must not be ignored given the fact that its prevalencehas increased; data from the literature suggests that as many as 78% of elderly have edentulism which can impact the health of other organs. It is well known that elderly people with dentures or dental pathology limit their intake of fruits and vegetables and consume large quantities of soft foods that are rich is saturated fat and cholesterol [5,6]. The reason why oral health gains worldwide attention is that it impacts the general health of individuals and their quality of life [7]. Given these reasons, an aging worldwide population requires the development of healthcare services that address the elderly pathology [8,9]. Among individuals that are 65 years old and above, the most frequent encountered dental pathologies include: periodontal disease, edentulism, dental caries, oral mucosal lesions, oral infections and temporomandibular pathology. Frequently among patients that take long-term medications for chronic pathology such as hypertension or dyslipidemia hyposalivation occurs, this greatly increases the risk for caries and mucosal infections [10,11]. The loosening of vertical dimension due to the age, the presence of bruxism or other oral parafunction that are very common in the elderly population can cause the patients to have a bad masticatory function, low quality of life and the risk of losing other teeth; it is also worth mentioning the high comorbidity index of elderly patients with numerous comorbidities such as coronary artery disease, neurological disease, diabetes mellitus that impact oral health [12,13,14].

The dental pathology can lead to chewing problems or a reduction in social interaction, therefore, in time nutritional deficiency can occurs or apparition of psychosocial distress. These problems are especially prevalent in elderly institutionalized patients [15,16].

The purpose of this narrative review is to present the most frequent dental pathology that affects the life of the elderly, to evaluate the impact of oral health among elderly on health and nutrition, to determine the level of access of elderly population to dental care services and to discuss the methods that can lead to improve the dental health of elderly.

## 2. Materials and Methods

We performed research of the relevant literature and selected the scientific publications addressing dental health pathology among elderly. The PubMed, Cochrane Library, Web of Science and Scopus databases were searched for relevant information related to this on the topic. The description of the whole process is illustrated in Table 1. The keywords mentioned at the beginning of this paper (“dental pathology elderly”, “dental care elderly”, “caries in older individuals”, “dental care access elderly” etc.). The articles considered eligible were initially selected based on both their title and abstract, then a complex analysis of their content was performed; the most relevant and informative data and results were extracted. Our article intended to present the particularities of dental pathology in elderly populations, certain barriers to dental care in this population and the necessary measures to overcome them. Given these objectives, we aimed to structure the presented information beginning from the requirements of an extensive and systematic review.

## 3. Oral Pathology in the Elderly Patient

### 3.1. Caries

Caries are the most frequent dental pathology encountered among the elderly; they are very prevalent because of salivary changes related to aging, the presence of xerostomia as a consequence of drug intake, the consumption of a poor diet, the exposure of dental roots because of gingival recession which occurs in the elderly and also the maintenance of teeth for a much longer period of time due to dental interventions [17]. In the elderly, it has been observed that there is a high prevalence of tooth extraction compared to younger population, where the more conservative approach of removing the least possible of the decayed tooth and maintain the natural teeth is chosen [18].

A study conducted in England demonstrated that the prevalence of caries in tooth crowns was 22%, and among those aged between 75 and 84 years-old, the active tooth decay was 20% [19]. In the United States, in a study performed in 2004, the index of caries experience defined as decayed, missing or filled teeth (DMFT) was 17.96; this was considered a high-index (−1.16). Among this index untreated dental caries represented a low proportion of 0.43, demonstrating that restorative services are frequently used among this population while the missing teeth component represented a high proportion of 8.81. Compared with previous years in the NHANES 1999–2004 analysis the DMFT index decreased, a phenomenon that occurred because of decrease in in missing teeth. In the same period of time, the number of decayed teeth and filled teeth increased (0.05). The index of decayed teeth and missing teeth where higher among the elderly of 75 years or above compared with the elderly aged between 65 and 77 years, while the index of filled teeth was lower in the age group of 75 years or older. These elements can be explained by the decreased access to dental care among the more elderly individuals [20].

Data from the study NHANES 2012 demonstrated a decrease in DMFT index as well as a high restorative index [21]. A similar analysis has been performed in the Fifth German Oral Health Survey [22]. Caries experience index DMFT in the age population of 65–74 years comparable to the one in the United States (DMFT 17.7), with a higher number of missing teeth (MT 11.1). Filled teeth indexed was high, while the number of decayed teeth was low (0.5). The missing teeth index decreased as the socioeconomical status of the individual increased [23]. In a study that analyzed the worldwide prevalence of dental caries among the elderly, this ranged from 25% in Australia to 99% in South Africa in community individuals. In China, the prevalence of dental caries was 98%, in India 77%, in USA 25%, in Finland 30%. The prevalence of root caries was 62% in China and 46% in India. The global median of mean prevalence of caries was 49%. The prevalence of untreated root caries ranged from 8% (Finland) to 74% (Brazil) in community dwellers [24].

### 3.2. Periodontal Disease

Periodontal disease that includes gingivitis and periodontitis is a disease of the tissues (periodontal attachment and bone) that support teeth [25]. Gingivitis refers to inflammation and bleeding of the gums and if left untreated, it will lead to periodontitis [26]. Periodontal disease was characterized as being the 11th most prevalent disease in the word by the Global Burden of Disease Study (2016) [27]. Bacterial plaque accumulation has been identified as an important cause of periodontal disease because it determines gingivitis and a process of mild or moderate alveolar bone loss [28]. A systematic review that included 37 countries determined that the incidence of periodontal disease was low among the elderly, but the prevalence of severe periodontitis increased with increasing age [29]. The bacterial factor may be the most important in the onset and severity of periodontitis, along with other factors such as the oral environment and the genetics of the host [30]. The first three periodontal pathogenic bacterial species, *Porphyromonas gingivalis*, *Tannerella forsythia*, and *Treponema denticola* were coined to the red complex [31]. Molecular biology studies on the subgingival bacterial complex initially identified the presence of 40 species and later 750 different species were identified in the oral cavity [32]. Another study analyzed the subgingival microbiota using NGS and identified 26 bacterial phyla, 433 genera, and 1016 species in the subgingival samples, it demonstrated the relationship between periodontal disease status and the subgingival microbiota. The study demonstrates that 4 dominant phyla, including Bacteroidetes, Fusobacteria, Synergistetes, and Spirochaetes, were found in periodontitis patients, and a remarkably high level of *P. gingivalis* in most subjects with periodontitis, whereas the level of *P. gingivalis* was relatively low in both healthy and gingivitis subjects, indicating that *P. gingivalis* is associated with periodontitis [33]. The genera Streptococcus, Haemophilus, and Leptotrichia might be correlated with gingivitis, as supported by the observation that these genera were 2-fold higher in gingivitis subjects than in healthy subjects [34]. Proteobacteria such as *H. hamiltonii*, *Moraxella osloensis*, *Acinetobacter junii*, and *Lautropia AP009*, were predominant in the microbiota of subgingival samples in healthy individuals [35]. The disruption of the bacterial balance in the oral cavity due to poor oral hygiene, change in diet, stress, chronic conditions growth of pathogenic bacteria and inhibition of healthy oral microbiota, can lead to periodontal disease [36,37,38].

Tooth loss and edentulism are the final stages of periodontal disease, this representing a failure of the dental care system. Edentulism is associated with nutritional deficits and significantly impacts the quality of life of the elderly; data from literature associates edentulism with increased mortality. Edentulism is highly associated in elderly population with the access to dental care services, offered by the state of public financial support or implementation of dental care policies [39,40,41]. Edentulism is highly prevalent around the world; 21.9% of patients over 74 in the United States of America and 39.6% from the same age group in New Zealand are affected by this condition. A peak of severe tooth loss in people 65 years old or above has been reported in developed and developing countries [42,43,44].

Periodontitis due to gingival retraction and exposure of tooth root can determine the apparition of caries. The development of caries is accompanied by xerostomia. Tooth retention is associated with increased number of visits at the dentist and with better quality of life [45]. Tooth loss and periodontitis represents a disability among the elderly, leading to poor mastication, poor nutritional choices, difficult speaking and psychological issues [46]. Tooth loss and periodontitis have been associated in an even larger context, leading to development of cardiovascular disease and diabetes mellitus [47,48].

The evolution of periodontist can be severe, and its treatment can be difficult because of tissue damage due to underlying systemic conditions [49,50]. In patients affected by this pathology a pocket depth if 5 mm or more has been correlated with a poor quality of life [51].

### 3.3. Oral Cancer in the Elderly

Oral cancers are more prevalent in male patients because they have a higher addiction to chewable tobacco which is a risk factor. Most studies demonstrate that the male to female ratio is 2:1, very few demonstrating an inverse relationship. The number and severity of comorbidities and the functional performance status in elderly patients significantly impact the therapeutic decision [52]. Among 310 evaluated elderly patients with head squamous cell cancer aged over 70 years, 75.1% had at least 1 comorbidity and in 13.9% of cases, this comorbidity was severe [53]. Oral cancer is an important cause or disability among adults and older populations in both high-income and low-income countries. Oral cancer includes lip cancer, oral cavity cancer, pharyngeal cancer and around the world is the eight most common type of cancer [54]. The treatment for oral cancer is surgical, and in the postoperative course can appear surgical complications, surgical infections and mortality. As demonstrated by data in the literature, older patients, although they had more comorbidities. the rate of complications was 23.2% vs. 20.2% in the younger population and 27.7% vs. 22.6% form minor complications [55]. The postoperative rate of infections as shown by another study in elderly patients was 33.7%, out of which 23.1% were surgical site infection and pneumonia was identified in 14.9% of patients [56]. In patients over 70 years old, survival was not significantly diminished compared to other age groups; however, more patients in this age group could not receive or complete appropriate adjuvant therapy. Therefore, oral cancer treatment needs to be individualized, keeping in mind the performance status of the patient and the comorbidities [57].

### 3.4. Edentulism

Edentulism is the final stage of untreated caries or periodontal disease [58]. Edentulism is prevalent worldwide; it has been reported that in elders over 74 years old, edentulism can be found in 21.9% of individuals in the United States of America, in Sweden the prevalence of edentulism among elders was 7% in 2000 [59,60]. The peak incidence of edentulism is around 65 years old as demonstrated by most studies in both developed and developing countries [61].

The prevalence of edentulism among general population is much lower than in the elders, a study performed in the United States on 432,519 adults demonstrated a prevalence of 4.9% [61]. Pelter evaluated the prevalence of edentulism in older individuals (50 years) in India, Russia, China, Ghana, Mexico and South Africa. The prevalence of edentulism was 16.3% in India, 9% in China, 21.7% in Russia and 8.5% prevalence in South Africa [62]. In Europe, many studies have evaluated the prevalence of edentulism, in Sweden the prevalence of this disease decreased markedly from 19% in 1975 to 3% in 1997 [63]. The causes of edentulism are complex. The main causes are diseases produced by the microbial detrimental role on the oral health, there is also interaction with the genetic profile of the individuals, also edentulism can appear as a consequence iatrogenic, traumatic or therapeutic cause [64].

The incidence of tooth loss was significantly higher among lower income and with poor education elders, and in elders with poor oral health or poor general health. A higher degree of periodontal disease is associated with history of smoking and low intake of ascorbic acid. The prevalence of complete edentulism differs between countries and within a country. It is extremely difficult to compare edentulism prevalence between nations because it can be affected by numerous factors [65].

In the United States of America, it is believed that the number of edentate individuals is 9 million, edentulism has a prevalence among individuals over 60 years old was 25% [66].

## 4. Impact of Oral Health among Elderly on Health and Nutrition

The “quality of life” includes a large a number of concepts such as health status, function and life conditions. Quality of life refers to the individual perception of one’s own health and this can be influenced by the culture and value system in which one lives. Perception of quality of live is different between individuals and it can modify over time [67]. Edentulism, as demonstrated by many studies, has a high impact on the quality of life [67,68,69]. Edentulism impacts day-to-day activities and social life. Oral function alteration causes a decreased self-esteem and reduces the psychosocial well-being. Elderly people with edentulism avoid social activities because they feel embarrassed to speak in front of other [70,71].

**Figure 1:**
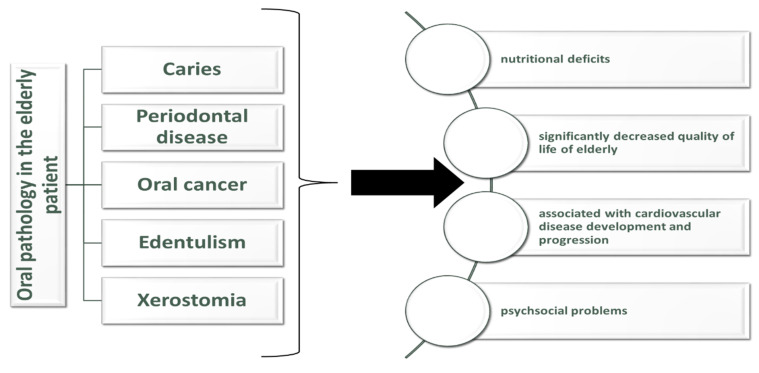
Oral health pathology in elderly and their consequences.

Data from the NHANES study confirms a strong association between oral health and general health (Figure 1. Oral health pathology in elderly and their consequences). Among persons with poor health, the prevalence of edentulism was 10% higher than among persons with good health. Complete or partial tooth loss were more prevalent among elderly people with chronic pathologies such as arthritis, low vision, coronary artery disease, chronic obstructive pulmonary disease. Individuals with untreated dental pathology reported the presence painful aching related to their dental pathology, this was reported in 32% of individuals aged between 50 and 64 years with poor health. Avoiding certain foods because of dental pathology was reported in 22% of elders aged between 65 and 74 years old and in 35% of elders aged 50 to 64 years old [72]. A lack of nutrients exacerbates the chronic pathology in the elderly and they can become predisposed to sarcopenia and frailty [73]. Anorexia can develop in older people because of the combination between acute or chronic illnesses and poor detention [74]. Dental pathology is associated with nutrient intake and with unhealthy eating habits that can increase the risk of diabetes and cardiovascular problems [75].

The number of teeth influences masticatory function, problems in functional occlusion, maximal biting force, denture wearing [76]. Tooth loss significantly impacts one’s chewing ability and makes bolus formation more difficult. If the number of teeth is reduced, then the bolus will increase in size and this determines a dysfunctional swallowing. Among edentulous elders, even in the one that use dentures, chewing is more difficult. This may cause these patients to change their diets [77]. Food deficiencies appear in older people with dental problems because they avoid harder foods such as meat, fruits or vegetables that are very reach in nutrients such as minerals, vitamins, protein, and fiber [78]. 

Nutritional deficits contribute to the development of sarcopenia. Lack of minerals and vitamins may contribute to oxidative stress and inflammation. These processes aggravate sarcopenia and also are risk factor for periodontitis [79]. Nutritional deficiencies reduce the mineralization process and this increases the risk for dental caries. Malnutrition also increases the severity of oral infections [80]. Oral health is a well-known risk factors for sarcopenia, psychological burden, cognitive impairment, and accumulating comorbidities [81].

Poor oral health increases the risk for oral infectious and non-infectious diseases. All of these elements contribute to the attention that the clinicians give to oral care. Poor oral health also increases the risk for mortality [82,83]. A community-based study conducted over 40 years demonstrated that the number of teeth is associated with all-cause mortality [84]. A systematic review demonstrated that the improvement of oral health protects against mortality; another study revealed that oral care during hospitalization of a patient can cause favorable mortality-outcomes [58,85]. Studies that evaluate the impact of oral health on mortality online include the dental problems related to teeth decay, periodontitis, gum disease, decreased number of teeth, however, poor oral health can refer to the condition of tongue, lips and saliva [86,87].

In a study performed on elders admitted in acute care hospital, elderly patients were divided in three groups according to the results of Oral Health Assessment Tool (OHAT) that was applied to these patients. The mean age of patients was 83.8 ± 7.9 years, they were divided in OHAT score equivalent to 0, 1–2 and ≥3, the mortality according to the OHAT score was 5.2%, 6.3% and 18.0%. It was observed that patients with OHAT score ≥3 had higher mortality than patients with lower OHAT scores, referring to patients with better oral health [88].

In a study that included a number of 344 elderly patients from Calabrian long-term facilities, the mean DMFT was 26.4. A GOHAI score ≤ 50 was reported in 1/3 of patients, this value representing a highly comprised oral health related quality of life. The GOHAI score was significantly higher in patients with lower DMFT. This demonstrates that a high degree of oral pathology severity can determine a poor quality of life [89]. A study conducted in Japan that included patients from 12 nursing homes analyzed by linear regression the association between dental conditions, comorbidities and sociodemographic elements. The study included 289 elderly patients, they had an average number of teeth of 11.6 and an average number of decayed teeth of 1.4. Dementia was significantly associated with increased risk of decayed teeth (*p* = 0.018). The presence of comorbidities such as hypertension, heart disease, arthritis was associated with the number of present teeth (*p* = 0.035, 0.005, and 0.023, respectively). It was observed that dementia was more prevalent in elders with more than 1 decayed teeth than in elders that did not have decayed teeth (*p* = 0.044) [90].

Another study identified in 198 elders with mean age of 81 years (±7.9) that 181 (91%) were dentate, the average teeth numbers was 19.2 (±6.5), 24% had teeth caries, 97% had filled teeth, 44% had <20 teeth and in 26% was reported oral dryness. DFT over the average was identified in patients suffering from COPD (*p* = 0.048), diabetes (*p* = 0.037), polypharmacy (*p* = 0.0111), diuretics (*p* = 0.007) and inhalation drugs (*p* = 0.032). The use of anticholinergic drugs was also associated with increased DFT (*p* = 0.004) [91].

## 5. Access of Elderly Patients to Oral Health Services

It is demonstrated that the elderly’s utilization of dental care is low, especially among those coming from low-income populations. The causes that were identified that lead to this fact are the inability to perceive the need to visit the dentist, fear, anxiety, past negative experiences, and lack of awareness toward the dental problems [92]. It is difficult to differentiate dental anxiety from lack of perceived need in older persons. The need for dental care is perceived only in persons who still have natural teeth, while edentulous individuals perceive no need of dental care. Dental professionals can offer to elders the adequate oral health education but only those who actively seek it [93,94].

A good oral health can prevent pathologies that cause a reduction in mastication and this can reduce the negative effects of malnutrition in old age [95]. In a study performed on 125 homebound elderly patients, it was revealed that among the total number of included patients, 61.0% last visited a dentist more than 3 years ago. Dental complaints included pain or discomfort in the oral cavity (33.6%), current tooth ache (15.1%), gum soreness (27.3%) and gum bleeding (16.3%). Chewing limitations were found in 50.4% of patients while 59.4% of dentures wearers were dissatisfied with their dentures. The individuals in the study had a poor oral health. The need of dental care in the elderly is very high and primary care physicians should have more attention regarding the dental problems of homebound, the medical community should cooperate with the dental community in order to develop home-based programs for older individuals [96].

Several measures have been proposed in order to increase the access of older people to dental care services. One of them was to include foreign domestic workers/caregivers intro the formal network, a fact that will decrease the pressure on the professionals. Improving knowledge regarding oral health among the elderly and policymakers is essential. Dental check-ups also should be included in the routine health care [97].

The management of older people’s dental care should be included in the development of undergraduate healthcare professionals. The National Working Group for Older People recommends that an extended consultation should be given to the elderly and a long-term oral health care plan tailored to their needs should be established. More dentists should be trained in order to care for the elderly and dental medicine curriculum should include lectures about elderly oral health [98,99].

**Figure 2:**
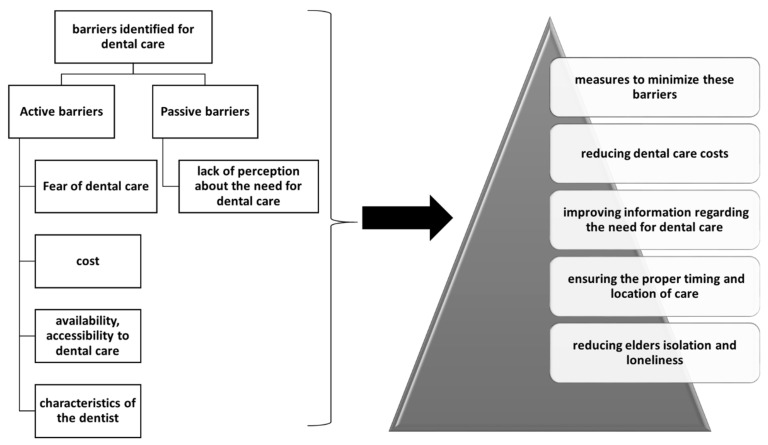
Barriers and methods to overcome them in dental care access of elderly.

In a study that included 39 older people and their healthcare providers, the active barriers to oral health care were evaluated. The main barriers identified for dental care activities in the elderly were: fear of medical intervention, cost of dental care, availability, accessibility and characteristics of the dentist (Figure 2. Barriers and methods to overcome them in dental care access of the elderly). A passive barrier was the lack of perception about the need for dental care. Additional barriers were the high cost of dental treatment or fear of dental treatment. Among the measures to minimize these barriers are: reducing dental care costs, improving information regarding the need for dental care, ensuring the proper timing and location of care, reducing elderly isolation and loneliness. Older patients cannot take personal initiative therefore they need the involvement of system and societal change [100].

During the COVID-19 pandemic, nursing homes improved their infection control protocols and dental care was deemed as a nonessential service. Dental care became for community-dwelling older adults and especially for those from nursing homes, one of the most postponed activities. The options for emergency dental care in nursing homes are limited. Tele dentistry is one of the options that makes possible the triage of dental pathology, referral to a hospital with dental medicine facilities for extractions or prescribing antibiotics [100,101].

During the pandemic in many states public health authorities recommended that emergency dental care to be performed only in private offices [102]. Dental care has become a high-risk activity for COVID-19 transmission because during this activity, aerosols that contain respiratory droplets are generated, and this is how the virus is transmitted [103].

In the future, new and enhanced infectious control precautions that target respiratory pathogens will need to be taken in dental clinics. These measures can include fogging with antiviral aerosols, the use of N95 masks, full-face shields, eye protection, isolation gowns and head covers, as well as filters in the dental office [104].

A sample of 350 elderly people has been investigated by face-to-face structured interviews regarding the access and utilization of dental care and the barriers to dental services. Among them, 37% had proper access to dental services. Independent variables that were associated with reduced access and utilization of dental services were community residents, unmarried participants, low income, smokers, irregular users of toothbrushing, no dental insurance and unaffordable price. In a logistic regression analysis, the factors associated with proper dental access were affordable prices, brushing regularly, higher education, being married [104]. Even in older studies, it has been demonstrated that costs significantly influence the patient’s decision to seek dental care. High costs are among one of the reasons for not going to the dentist [105,106]. Wealth and income have a strong positive impact on dental care (95). Dental insurance coverage also increases the dental care use. Dental care insurance significantly differs according to the country ranging from the highest values in Germany (98%), Czech Republic (96%) to middles coverage in USA (48%) and Spain (31%) and to the lowest values in Switzerland (21%) and Italy (21%) [107]. In older age, the frequency of dental visits decreases, this happens because of non-modifiable factors such as aging and worsening of general health or of modifiable factors such as change in weight category [108].

In the United States, there are disparities in the prevalence of edentulousness varies according to the socio-economic and educational status. In 2018, the overall rate of edentulousness was 12.9% for persons aged 65 and older. Edentulousness was about 40% in low-income older adults double than in higher-income older adults [109]. In the United States, dental care is considered rather a privilege, this being a reflection of the culture that places on the first-place individualism. Each state is responsible for its oral healthcare policies, although there exists federal support. Payment for dental care is out-of-pocket, only 29.2% of elders have dental health insurance and this percentage declines with age [110].

## 6. Methods to Improve Oral Health among Elderly

Oral health promotion refers to increase the knowledge of dental practitioners regarding the dental problems of older adults. It also encourages screening for dental pathology in elders for early identification of tooth decay or periodontal disease. Columbia University College of Dental Medicine initiated a community-based outreach program of preventive and referral services named ElderSmile. The elders included in ElderSmile required almost all of them dental treatment, the majority who were screened followed up with treatment at neighborhood sites [111].

In Australia, Nursing Home Oral and Dental Health Plan was implemented in 2010. The plan included a national evidence based oral health model called Better Oral Health in Residential Care. Dental professionals offered their service to patients from these nursing homes. The causes that reduce the care of elders from residential aged care facilities by dental professionals are reduced clinical time in practice, difficulty in providing clinical care in a non-dental environment and lack of referral pathways from the residential aged care facilities to the dentists. Minimal intervention with glass ionomer cement and silver fluoride was used. The model emphasizes the use of palliative and definitive treatment in elder patients [112].

Encouragement of flossing was associated with better oral heal among the elderly. Data from Piedmont 65+ Dental Study demonstrated that elderly flossers had lower interproximal clinical attachment level (iCAL) and interproximal probing depth (iPD) compared to non-flossers (*p* ≤ 0.005). Elders who floss had statically significant less coronal caries compared to non-flossers (*p* = 0.02). Baseline number of missing teeth (mean, SE) was 11.5 (0.35) in non-flossers was statistically significant higher compared to 8.6 (0.53) in flossers (*p* < 0.0001). Regular dental visitors had lower oral disease levels compared to episodic dental user. At the 5-y follow-up visit, the average tooth loss for flossers was ~1 tooth significantly lower compared to ~4 teeth lost for non-flossers (*p* < 0.0001). Among all teeth, molars showed the highest benefit (>40%) of flossing behavior (*p* = 0.0005). These results further support flossing as an important oral hygiene behavior to prevent dental pathology progression in older adults [113].

There are different methods to improve the dental care of the elderly. One of the methods includes a public health campaign that should promote the importance of oral health among the elderly. The other includes creating a coalition of professionals from legislative and executive branches in order to implement programs for dental care. Another option consists in implementing a set of financial options for dental care that include essential benefits and supports quality care in order to reduce the costs. The private-sector insurers should be involved as well in analyzing the befits of dental programs especially in analyzing the befits of oral health care programs and the correlation between disease severity and costs need for oral health care. Moreover, new models for dental care delivery should be used such as mobile technology, tele-dentistry, adoption of oral health teams, and integration with geriatric and primary care offer opportunities [114].

**Figure 3:**
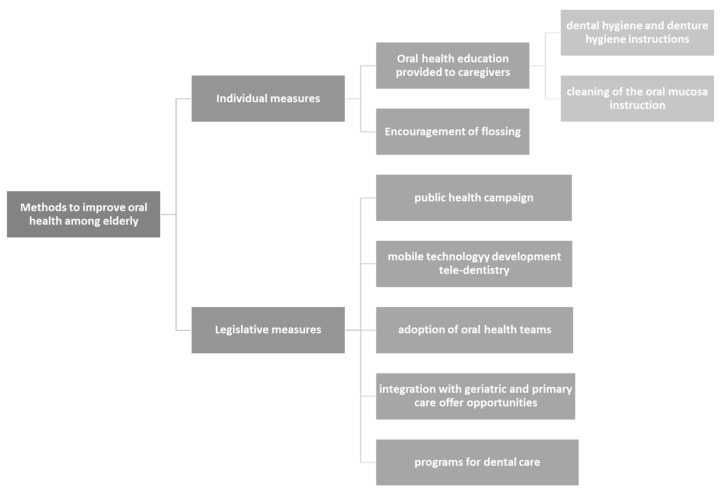
Methods to improve dental care among elderly.

A meta-analysis includes eighty-one studies that evaluated the impact of oral health care interventions. Oral health care interventions included: educational interventions, providing oral hygiene and assistance with toothbrushing, application of fluoride, mouthwashes, telemedicine, probiotics (Figure 3. Methods to improve dental care among the elderly). Most interventions on the care/patient level (56/81) and on the system/policy-maker (44/81). Primary interventions were the most common. Given the heterogeneity of the interventions no concussions could be drawn. The meta-analysis remarked the lack of early interventions measures included in secondary interventions [115].

Oral health education provided to caregivers is important, it includes improving the knowledge about oral health, dental hygiene, oral health, denture care, dental check-ups. One study evaluating such interventions demonstrates that postintervention caretakers had improved knowledge about oral health oral hygiene, denture care, use of fluorides and importance of regular dental check-ups [116,117,118,119]. 

A study that evaluated the impact of dental health intervention included 151 patients in the intervention group and 181 in the control groups. The intervention consisted in the following instructions: dental hygiene instructions, denture hygiene instructions and cleaning of the oral mucosa instruction. The instructions were given to the patient or to the caregiver. The participants were examined after 6 months from the beginning of the study in order to assess the dental health. Patients from the intervention group had a significant reduction in the number of teeth with plaque (Estimate 2.6, 95% CI: 0.3; 4.8) and improvement on cleanliness of dentures (OR 2.1, 95% CI: 0.7; 3.4) Those who brushed their teeth less than twice daily had higher number of plaque teeth (Estimate 2.7, 95% CI: 0.3; 5.1). At the beginning of the study, 17 participants (15%) in the intervention group declared problems in cleaning the mouth and dentures, mainly due to reduced dexterity, reduced cognition and pain. After the intervention, this proportion increased to 21% (*n* = 28). In the control group, 20 participants (19%) reported the same problems (mainly decreased dexterity) in daily hygiene during both examinations [117]. Table 2 depicts the main findings of systematic reviews that analyzed the impact of certain interventions for improving oral health among the elderly. 

The strong point of the study consists in the ample manner of the presentation of oral health pathology and especially the many levels where it impacts the general health of this category of population with a great reduction in general health. The review also presents details about the reasons why elders do not access oral health care services. Another strong point of the study consists in the exposure of certain measures that can be taken for the individual elder or the elderly community in order to increase the utilization of these services and prevent further progression of dental pathology. The review is a narrative one and this could be considered a limitation of the study compared to a meta-analysis, however, because it is an extensive and systematic review, the reader can gain an insight of the many faces of a complex thematic, the neglected dental pathology of the elders. 

## 7. Conclusions

Dental pathology is complex among the elderly and as shown in the literature impact the general health by inducing nutritional deficiencies, sarcopenia, weakening of the immune system and increasing the cardiovascular risk. Numerous barriers, both active and passive, related to dental care can be encountered in this population costs being among them and lack of awareness by the elders of their dental pathology. Healthcare policymakers must implement programs that educate the elderly and the caregivers to improve dental hygiene and refer the elder to a specialist but also dentist must be more informed about the importance of dental health in this category of population. Primary and secondary prevention programs need to be implemented since data and information from the literature demonstrates their efficacy in the prevention of edentulism, tooth decay, periodontitis and improving quality of life. A collaboration between primary caregivers and dentist is essential in achieving these goals. 

## Figures and Tables

**Table 1:** Metodology for bibliography selection.

Identification of Studies via Databases and Registers for Realisation of the Extensive Review		
Identification	Key words with OR operator	“dental pathology elderly”, “dental care elderly”, “caries in older individuals”, “dental care access elderly”
	Consulted Databases	Web of Science, PubMed, Cochrane, Scopus
	Criteria for inclusion	Review or Original Article, cited at least 20 times, relevant for the topic and thematic, articles that focus on elderly people pathology
	Criteria for exclusion	Abstract paper, articles cited less than 20 times, not relevant for the topic and thematic, articles that did not focus on elderly pathology, misleading title related to content
Records identified	Web of Science	145 papers: 116 original papers + 29 review papers
	Pubmed	126 papers: 110 original papers + 16 review papers
	Cochrane	39 papers: 32 original papers + 7 review papers
	Scopus	50 papers: 38 original + 12 review papers
Total records	Web of Science + Pubmed + Cochrane + Scopus	350 papers: 296 original papers + 64 review papers
Duplicates removal	Web of Science + Pubmed + Cochrane + Scopus	exclusion of 83 papers
Full-text analysis	Appliance of inclusion and exclusion criteria	exclusion 145 papers
Final bibliographical source		122 papers: 88 original papers + 2 review papers

**Table 2:** Main findings of systematic reviews that analyzed the impact of certain interventions for improving oral health among elderly.

Authors	Number of Included Studies	Type of Study	Main Findings	Reference
McGrath C et al.	17	Systematic Review	Fluoride use, antimicrobial agents use and health-care provider education are important measures for oral health promotion activities for elderly people	[120]
Manchery N et al.	4	Systematic Review	Significant improvement of certain oral health measures in dementia elderly following a carer oral health education program	[121]
Nakre P et al.	40	Systematic Review	Oral health education was effective in improving the knowledge attitude and practice of oral health among elderly and in reducing bleeding on probing, plaque index and caries progression	[122]
Gommez-Rossi J et al.	81	Systematic Review	Most studies (64/81) found a statistically significant benefit of the intervention in elderly. Numerous intervention types employed (e.g., professional oral healthcare, educational interventions, restorative treatment, fluoride application and, generally, dentifrices, mouthwashes, chewing gums/food supplements)	[117]

## References

[1] Cho E.-P., Hwang S.-J., Clovis J.B., Lee T.-Y., Paik D.-I., Hwang Y.-S. (2012). Enhancing the Quality of Life in Elderly Women through a Programme to Improve the Condition of Salivary Hypofunction: Oral Health Promotion Programme and Quality of Life. Gerodontology.

[2] Glazar I., Urek M.M., Brumini G., Pezelj-Ribaric S. (2010). Oral Sensorial Complaints, Salivary Flow Rate and Mucosal Lesions in the Institutionalized Elderly. J. Oral. Rehabil..

[3] Sischo L., Broder H.L. (2011). Oral Health-Related Quality of Life: What, Why, How, and Future Implications. J. Dent. Res..

[4] Cosola S., Marconcini S., Giammarinaro E., Poli G.L., Covani U., Barone A. (2018). Oral Health-Related Quality of Life and Clinical Outcomes of Immediately or Delayed Loaded Implants in the Rehabilitation of Edentulous Jaws: A Retrospective Comparative Study. Minerva Dent. Oral Sc..

[5] De Lima Saintrain M.V., Gonçalves R.D. (2013). Salivary Tests Associated with Elderly People’s Oral Health: Salivary Test in Elderly People. Gerodontology.

[6] Kossioni A.E., Dontas A.S. (2007). The Stomatognathic System in the Elderly. Useful Information for the Medical Practitioner. Clin. Interv. Aging.

[7] World Health Organization (WHO) Oral Health. https://www.euro.who.int/en/health-topics/disease-prevention/oral-health#.

[8] De Oliveira T.C., da Silva D.A., Leite de Freitas Y.N., da Silva R.L., Pegado C.P.d.C., de Lima K.C. (2013). Socio-Demographic Factors and Oral Health Conditions in the Elderly: A Population-Based Study. Arch. Gerontol. Geriatr..

[9] Tsakos G. (2011). Inequalities in Oral Health of the Elderly: Rising to the Public Health Challenge?. J. Dent. Res..

[10] Chalmers J., Pearson A. (2005). Oral Hygiene Care for Residents with Dementia: A Literature Review. J. Adv. Nurs..

[11] Jokanovic N., Tan E.C.K., Dooley M.J., Kirkpatrick C.M., Bell J.S. (2015). Prevalence and Factors Associated with Polypharmacy in Long-Term Care Facilities: A Systematic Review. J. Am. Med. Dir. Assoc..

[12] Goldstein G., Goodacre C., MacGregor K. (2021). Occlusal Vertical Dimension: Best Evidence Consensus Statement. J. Prosthodont..

[13] Morita K., Kimura H., Tsuka H., Nishio F., Yoshida M., Tsuga K. (2020). Association between Salivary Alpha-Amylase and Subjective and Objective Oral Parafunctions in Community-Dwelling Elderly Individuals. J. Dent. Sci..

[14] Bondar A., Popa A.R., Papanas N., Popoviciu M., Vesa C.M., Sabau M., Daina C., Stoica R.A., Katsiki N., Stoian A.P. (2021). Diabetic Neuropathy: A Narrative Review of Risk Factors, Classification, Screening and Current Pathogenic Treatment Options (Review). Exp. Ther. Med..

[15] Bekiroglu N., Çiftçi A., Bayraktar K., Yavuz A., Kargul B. (2012). Oral Complaints of Denture-Wearing Elderly People Living in Two Nursing Homes in Istanbul, Turkey. Oral. Health Dent. Manag..

[16] Razak P.A., Richard K.M.J., Thankachan R.P., Hafiz K.A.A., Kumar K.N., Sameer K.M. (2014). Geriatric Oral Health: A Review Article. J. Int. Oral. Health.

[17] Wyatt C.C.L., Wang D., Aleksejuniene J. (2014). Incidence of Dental Caries among Susceptible Community-Dwelling Older Adults Using Fluoride Toothpaste: 2-Year Follow-up Study. J. Can. Dent. Assoc..

[18] Hayes M., Allen E., da Mata C., McKenna G., Burke F. (2014). Minimal Intervention Dentistry and Older Patients Part 2: Minimally Invasive Operative Interventions. Dent. Update.

[19] Thomson W.M. (2004). Dental Caries Experience in Older People over Time: What Can the Large Cohort Studies Tell Us?. Br. Dent. J..

[20] Dye B.A., Tan S., Smith V., Lewis B.G., Barker L.K., Thornton-Evans G., Eke P.I., Beltrán-Aguilar E.D., Horowitz A.M., Li C.-H. (2007). Trends in Oral Health Status: United States, 1988–1994 and 1999–2004.

[21] Dye B., Thornton-Evans G., Li X., Iafolla T. (2015). Dental Caries and Tooth Loss in Adults in the United States, 2011–2012. NCHS Data Brief..

[22] Jordan R.A., Micheelis W. (2015). Krankheitsund Versorgungspravalenzen Bei Alteren Senioeren Mit Pflegebedarf.

[23] Schiffner U. (2015). Krankheits- Und Versorgungspravalenzen Bei Jungeren Senioeren (65–74-Jahrige): Karies Und Erosionen.

[24] Chan A.K.Y., Tamrakar M., Jiang C.M., Lo E.C.M., Leung K.C.M., Chu C.H. (2021). A Systematic Review on Caries Status of Older Adults. Int. J. Environ. Res. Public Health.

[25] Newman M.G. (2011). Carranza’s Clinical Periodontology.

[26] World Health Organization (2018). Oral Health.

[27] Vos T., Abajobir A.A., Abate K.H., Abbafati C., Abbas K.M., Abd-Allah F., Abdulkader R.S., Abdulle A.M., Abebo T.A., Abera S.F. (2017). Global, Regional, and National Incidence, Prevalence, and Years Lived with Disability for 328 Diseases and Injuries for 195 Countries, 1990–2016: A Systematic Analysis for the Global Burden of Disease Study 2016. Lancet.

[28] Hirotomi T., Yoshihara A., Yano M., Ando Y., Miyazaki H. (2002). Longitudinal Study on Periodontal Conditions in Healthy Elderly People in Japan. Community Dent. Oral Epidemiol..

[29] Kassebaum N.J., Bernabé E., Dahiya M., Bhandari B., Murray C.J.L., Marcenes W. (2014). Global Burden of Severe Periodontitis in 1990-2010: A Systematic Review and Meta-Regression. J. Dent. Res..

[30] Albandar J.M. (2005). Epidemiology and risk factors of periodontal diseases. Dent. Clin. N. Am..

[31] Socransky S.S., Haffajee A.D., Cugini M.A., Smith C., Kent R.L. (1998). Microbial complexes in subgingival plaque. J. Clin. Microbiol..

[32] Abusleme L., Dupuy A.K., Dutzan N., Silva N., Burleson J.A., Strausbaugh L.D., Gamonal J., Diaz P.I. (2013). The subgingival microbiome in health and periodontitis and its relationship with community biomass and inflammation. ISME J..

[33] Park O.J., Yi H., Jeon J.H., Kang S.S., Koo K., Kum K.Y., Chun J., Yun C.H., Han S.H. (2015). Pyrosequencing Analysis of Subgingival Microbiota in Distinct Periodontal Conditions. J. Dent. Res..

[34] Huang S., Yang F., Zeng X., Chen J., Li R., Wen T., Li C., Wei W., Liu J., Chen L. (2011). Preliminary characterization of the oral microbiota of Chinese adults with and without gingivitis. BMC Oral Health.

[35] Griffen A.L., Beall C.J., Campbell J.H., Firestone N.D., Kumar P.S., Yang Z.K., Podar M., Leys E.J. (2012). Distinct and complex bacterial profiles in human periodontitis and health revealed by 16S pyrosequencing. ISME J..

[36] Shaddox L.M., Walker C.B. (2010). Treating chronic periodontitis: Current status, challenges, and future directions. Clin. Cosmet. Investig. Dent..

[37] Darveau R.P. (2010). Periodontitis: A polymicrobial disruption of host homeostasis. Nat. Rev. Microbiol..

[38] Zhang Y., Wang X., Li H., Ni C., Du Z., Yan F. (2018). Human oral microbiota and its modulation for oral health. Biomed. Pharmacother..

[39] Kotronia E., Brown H., Papacosta A.O., Lennon L.T., Weyant R.J., Whincup P.H., Wannamethee S.G., Ramsay S.E. (2021). Oral Health and All-Cause, Cardiovascular Disease, and Respiratory Mortality in Older People in the UK and USA. Sci. Rep..

[40] Brown D.W. (2009). Complete Edentulism Prior to the Age of 65 Years Is Associated with All-Cause Mortality. J. Public Health Dent..

[41] Emami E., de Souza R.F., Kabawat M., Feine J.S. (2013). The Impact of Edentulism on Oral and General Health. Int. J. Dent..

[42] Ministry of Health (2010). Our Oral Health: Key Findings of the 2009 New Zealand Oral Health Survey.

[43] Gerritsen A.E., Allen P.F., Witter D.J., Bronkhorst E.M., Creugers N.H.J. (2010). Tooth Loss and Oral Health-Related Quality of Life: A Systematic Review and Meta-Analysis. Health Qual. Life Outcomes.

[44] Kassebaum N.J., Bernabé E., Dahiya M., Bhandari B., Murray C.J.L., Marcenes W. (2014). Global Burden of Severe Tooth Loss: A Systematic Review and Meta-Analysis: A Systematic Review and Meta-Analysis. J. Dent. Res..

[45] Somsak K., Kaewplung O. (2015). The Effects of the Number of Natural Teeth and Posterior Occluding Pairs on the Oral Healthrelated Quality of Life in Elderly Dental Patients. Gerodontology.

[46] Roohafza H., Afghari P., Keshteli A.H., Vali A., Shirani M., Adibi P., Afshar H. (2015). The Relationship between Tooth Loss and Psychological Factors. Community Dent. Health.

[47] Liljestrand J.M., Havulinna A.S., Paju S., Männistö S., Salomaa V., Pussinen P.J. (2015). Missing Teeth Predict Incident Cardiovascular Events, Diabetes, and Death. J. Dent. Res..

[48] Padilha D.M.P., Hilgert J.B., Hugo F.N., Bós A.J.G., Ferrucci L. (2008). Number of Teeth and Mortality Risk in the Baltimore Longitudinal Study of Aging. J. Gerontol. A Biol. Sci. Med. Sci..

[49] Ehizele A., Ojehanon P. (2016). Periodontal Conditions Seen in a Group of Nigerian Older Adult Patients. J. Interdiscip. Dent..

[50] Renvert S., Persson G.R. (2016). Treatment of Periodontal Disease in Older Adults. Periodontology 2000.

[51] Eltas A., Uslu M.O., Eltas S.D. (2016). Association of Oral Health-Related Quality of Life with Periodontal Status and Treatment Needs. Oral Health Prev. Dent..

[52] Sarini J., Fournier C., Lefebvre J.L., Bonafos G., Van J.T., Coche-Dequéant B. (2001). Head and Neck Squamous Cell Carcinoma in Elderly Patients: A Long-Term Retrospective Review of 273 Cases. Arch. Otolaryngol. Head Neck Surg..

[53] Sanabria A., Carvalho A.L., Vartanian J.G., Magrin J., Ikeda M.K., Kowalski L.P. (2007). Comorbidity Is a Prognostic Factor in Elderly Patients with Head and Neck Cancer. Ann. Surg. Oncol..

[54] Petersen P.E., Bourgeois D., Ogawa H., Estupinan-Day S., Ndiaye C. (2005). The global burden of oral diseases and risk to oral health. Bull. World Health Organ..

[55] Gugić J., Strojan P. (2012). Squamous Cell Carcinoma of the Head and Neck in the Elderly. Rep. Pract. Oncol. Radiother..

[56] Paleri V., Wight R.G., Silver C.E., Haigentz M., Takes R.P., Bradley P.J., Rinaldo A., Sanabria A., Bień S., Ferlito A. (2010). Comorbidity in Head and Neck Cancer: A Critical Appraisal and Recommendations for Practice. Oral Oncol..

[57] Malik A., Mishra A., Chopda P., Singhvi H., Nair S., Nair D., Laskar S.G., Prabhash K., Agarwal J.P., Chaturvedi P. (2019). Impact of Age on Elderly Patients with Oral Cancer. Eur. Arch. Otorhinolaryngol..

[58] Polzer I., Schwahn C., Völzke H., Mundt T., Biffar R. (2012). The Association of Tooth Loss with All-Cause and Circulatory Mortality. Is There a Benefit of Replaced Teeth? A Systematic Review and Meta-Analysis. Clin. Oral. Investig..

[59] Dye B.A., Li X., Thorton-Evans G. (2012). Oral Health Disparities as Determined by Selected Healthy People 2020 Oral Health Objectives for the United States, 2009–2010.

[60] Osterberg T., Carlsson G.E. (2007). Dental State, Prosthodontic Treatment and Chewing Ability - a Study of Five Cohorts of 70-Year-Old Subjects. J. Oral Rehabil..

[61] Fantin R., Delpierre C., Kelly-Irving M., Barboza Solís C. (2018). Early socioeconomic conditions and severe tooth loss in middle-aged Costa Ricans. Community Dent Oral Epidemiol..

[62] Slade G.D., Akinkugbe A.A., Sanders A.E. (2014). Projections of US Edentulism Prevalence Following 5 Decades of Decline. J. Dent. Res..

[63] Peltzer K., Hewlett S., Yawson A.E., Moynihan P., Preet R., Wu F., Guo G., Arokiasamy P., Snodgrass J.J., Chatterji S. (2014). Prevalence of Loss of All Teeth (Edentulism) and Associated Factors in Older Adults in China, Ghana, India, Mexico, Russia and South Africa. Int. J. Environ. Res. Public Health.

[64] Osterberg T., Carlsson G.E., Sundh V. (2000). Trends and Prognoses of Dental Status in the Swedish Population: Analysis Based on Interviews in 1975 to 1997 by Statistics Sweden. Acta Odontol. Scand..

[65] Cooper L.F. (2009). The Current and Future Treatment of Edentulism. J. Prosthodont..

[66] Müller F., Naharro M., Carlsson G.E. (2007). What Are the Prevalence and Incidence of Tooth Loss in the Adult and Elderly Population in Europe?. Clin. Oral. Implants Res..

[67] Beltrán-Aguilar E.D., Barker L.K., Canto M.T. (2005). Surveillance for Dental Caries, Dental Sealants, Tooth Retention, Edentulism, and Enamel Fluorosis—United States, 1988–1994 and 1999–2002.

[68] Seerig L.M., Nascimento G.G., Peres M.A., Horta B.L., Demarco F.F. (2015). Tooth loss in adults and income: Systematic review and meta-analysis. J Dent..

[69] Hugo F.N., Hilgert J.B., da Luz Rosário de Sousa M., Cury J.A. (2009). Oral Status and Its Association with General Quality of Life in Older Independent-Living South-Brazilians. Community Dent. Oral Epidemiol..

[70] Nitschke I., Müller F. (2004). The Impact of Oral Health on the Quality of Life in the Elderly. Oral Health Prev. Dent..

[71] Heydecke G., Thomason J.M., Lund J.P., Feine J.S. (2006). The Impact of Conventional and Implant Supported Prostheses on Social and Sexual Activities in Edentulous Adults: Results from a Randomized Trial 2 Months after Treatment. J. Prosthet. Dent..

[72] Rodrigues S.M., Oliveira A.C., Vargas A.M.D., Moreira A.N., E Ferreira E.F. (2012). Implications of Edentulism on Quality of Life among Elderly. Int. J. Environ. Res. Public Health.

[73] (2008). National Center for Health Statistics National Health and Nutrition Examination Survey Data [2003–2008].

[74] Morley J.E. (2017). Anorexia of Ageing: A Key Component in the Pathogenesis of Both Sarcopenia and Cachexia: Editorial. J. Cachexia Sarcopenia Muscle.

[75] Agarwal E., Miller M., Yaxley A., Isenring E. (2013). Malnutrition in the Elderly: A Narrative Review. Maturitas.

[76] Touger-Decker R. (2010). Diet, Cardiovascular Disease and Oral Health. J. Am. Dent. Assoc..

[77] Naka O., Anastassiadou V., Pissiotis A. (2014). Association between Functional Tooth Units and Chewing Ability in Older Adults: A Systematic Review. Gerodontology.

[78] Greksa L.P., Parraga I.M., Clark C.A. (1995). The Dietary Adequacy of Edentulous Older Adults. J. Prosthet. Dent..

[79] Hung H.-C., Colditz G., Joshipura K.J. (2005). The Association between Tooth Loss and the Self-Reported Intake of Selected CVD-Related Nutrients and Foods among US Women. Community Dent. Oral Epidemiol..

[80] Takahashi M., Maeda K., Wakabayashi H. (2018). Prevalence of Sarcopenia and Association with Oral Health-Related Quality of Life and Oral Health Status in Older Dental Clinic Outpatients. Geriatr. Gerontol. Int..

[81] Friedlander A.H., Weinreb J., Friedlander I., Yagiela J.A. (2007). Metabolic Syndrome: Pathogenesis, Medical Care and Dental Implications. J. Am. Dent. Assoc..

[82] Gil-Montoya J.A., Sánchez-Lara I., Carnero-Pardo C., Fornieles-Rubio F., Montes J., Barrios R., Gonzalez-Moles M.A., Bravo M. (2017). Oral Hygiene in the Elderly with Different Degrees of Cognitive Impairment and Dementia. J. Am. Geriatr. Soc..

[83] Almirall J., Serra-Prat M., Bolíbar I., Balasso V. (2017). Risk Factors for Community-Acquired Pneumonia in Adults: A Systematic Review of Observational Studies. Respiration.

[84] Lee J.-H., Oh J.-Y., Youk T.-M., Jeong S.-N., Kim Y.-T., Choi S.-H. (2017). Association between Periodontal Disease and Non-Communicable Diseases: A 12-Year Longitudinal Health-Examinee Cohort Study in South Korea. Medicine.

[85] Jansson L., Kalkali H., Mulk Niazi F. (2018). Mortality Rate and Oral Health—A Cohort Study over 44 Years in the County of Stockholm. Acta Odontol. Scand..

[86] Sjögren P., Wårdh I., Zimmerman M., Almståhl A., Wikström M. (2016). Oral Care and Mortality in Older Adults with Pneumonia in Hospitals or Nursing Homes: Systematic Review and Meta-Analysis. J. Am. Geriatr. Soc..

[87] Nakashima T., Maeda K., Tahira K., Taniguchi K., Mori K., Kiyomiya H., Akagi J. (2018). Silent Aspiration Predicts Mortality in Older Adults with Aspiration Pneumonia Admitted to Acute Hospitals: Silent Aspiration in Pneumonia. Geriatr. Gerontol. Int..

[88] Chalmers J.M., King P.L., Spencer A.J., Wright F.A.C., Carter K.D. (2005). The Oral Health Assessment Tool—Validity and Reliability. Aust. Dent. J..

[89] Maeda K., Mori N. (2020). Poor Oral Health and Mortality in Geriatric Patients Admitted to an Acute Hospital: An Observational Study. BMC Geriatr..

[90] Bianco A., Mazzea S., Fortunato L., Giudice A., Papadopoli R., Nobile C.G.A., Pavia M. (2021). Oral Health Status and the Impact on Oral Health-Related Quality of Life among the Institutionalized Elderly Population: A Cross-Sectional Study in an Area of Southern Italy. Int. J. Environ. Res. Public Health.

[91] Ide K., Seto K., Usui T., Tanaka S., Kawakami K. (2018). Correlation between Dental Conditions and Comorbidities in an Elderly Japanese Population: A Cross-Sectional Study. Medicine.

[92] Andersson P., Kragh Ekstam A. (2021). Impaired Oral Health in Older Orthopaedic In-Care Patients: The Influence of Medication and Morbidity. Clin. Interv. Aging.

[93] Petersen P.E., Yamamoto T. (2005). Improving the Oral Health of Older People: The Approach of the WHO Global Oral Health Programme. Community Dent. Oral Epidemiol..

[94] Liddell A., Locker D. (1993). Dental Anxiety in the Elderly. Psychol. Health.

[95] Slack-Smith L., Lange A., Paley G., O’Grady M., French D., Short L. (2009). Oral Health and Access to Dental Care: A Qualitative Investigation among Older People in the Community: Oral Health and Access to Dental Care. Gerodontology.

[96] Metcalf S.S., Northridge M.E., Lamster I.B. (2011). A Systems Perspective for Dental Health in Older Adults.

[97] Ornstein K.A., DeCherrie L., Gluzman R., Scott E.S., Kansal J., Shah T., Katz R., Soriano T.A. (2015). Significant Unmet Oral Health Needs of Homebound Elderly Adults. J. Am. Geriatr. Soc..

[98] Nitschke I., Stillhart A., Kunze J. (2015). Utilization of Dental Services in Old Age. Swiss Dent. J..

[99] Borreani E., Wright D., Scambler S., Gallagher J.E. (2008). Minimising Barriers to Dental Care in Older People. BMC Oral Health.

[100] Gallagher J.E., Fiske J. (2007). Special Care Dentistry: A Professional Challenge. Br. Dent. J..

[101] Rocha J.S., Arima L., Chibinski A.C., Werneck R.I., Moysés S.J., Baldani M.H. (2018). Barriers and facilitators to dental care during pregnancy: A systematic review and meta-synthesis of qualitative studies. Cad Saude Publ..

[102] American Geriatrics Society (2020). American Geriatrics Society Policy Brief: COVID-19 and Nursing Homes: Ags Policy Brief: COVID-19 & Nursing Homes. J. Am. Geriatr. Soc..

[103] Centers for Medicare & Medicaid Services CMS Releases Recommendations on Adult Elective Surgeries, Non-Essential Medical, Surgical, And Dental Procedures during COVID-19 Response. https://www.cms.gov/newsroom/press-releases/cms-releases-recommendations-adult-elective-surgeries-non-essential-medical-surgical-and-dental.

[104] Ren Y.F., Rasubala L., Malmstrom H., Eliav E. (2020). Dental Care and Oral Health under the Clouds of COVID-19. JDR Clin. Trans. Res..

[105] Meng L., Hua F., Bian Z. (2020). Coronavirus Disease 2019 (COVID-19): Emerging and Future Challenges for Dental and Oral Medicine. J. Dent. Res..

[106] Hamasha A.-H., Aldosari M., Alturki A., Aljohani S., Aljabali I., Alotibi R. (2019). Barrier to Access and Dental Care Utilization Behavior with Related Independent Variables in the Elderly Population of Saudi Arabia. J. Int. Soc. Prev. Community Dent..

[107] Bomberg T.J., Ernst N.S. (1986). Improving Utilization of Dental Care Services by the Elderly. Gerodontics.

[108] Conrad D.A. (1983). Dental Care Demand: Age-Specific Estimates for the Population 65 Years of Age and Over. Health Care Financ. Rev..

[109] Manski R., Moeller J., Chen H., Widström E., Lee J., Listl S. (2015). Disparity in Dental Coverage among Older Adult Populations: A Comparative Analysis across Selected European Countries and the USA. Int. Dent. J..

[110] Spinler K., Aarabi G., Valdez R., Kofahl C., Heydecke G., König H.-H., Hajek A. (2019). Prevalence and Determinants of Dental Visits among Older Adults: Findings of a Nationally Representative Longitudinal Study. BMC Health Serv. Res..

[111] Fleming E., Afful J., Griffin S.O. (2020). Prevalence of tooth loss among older adults: United States, 2015-2018. NCHS Data Brief..

[112] Kramarow E.A. (2019). Dental Care among Adults Aged 65 and over, 2017. NCHS Data Brief..

[113] Marshall S., Northridge M.E., De La Cruz L.D., Vaughan R.D., O’Neil-Dunne J., Lamster I.B. (2009). ElderSmile: A Comprehensive Approach to Improving Oral Health for Seniors. Am. J. Public Health.

[114] Lewis A., Wallace J., Deutsch A., King P. (2015). Improving the Oral Health of Frail and Functionally Dependent Elderly. Aust. Dent. J..

[115] Marchesan J.T., Byrd K.M., Moss K. (2020). Flossing Is Associated with Improved Oral Health in Older Adults. J. Dent. Res..

[116] Raphael C. (2017). Oral Health and Aging. Am. J. Public Health.

[117] Gomez-Rossi J., Hertrampf K., Abraham J., Gaßmann G., Meyer G., Schlattmann P., Göstemeyer G., Schwendicke F. (2020). Interventions to Improve Oral Health of Older People: A Scoping Review. J. Dent..

[118] Khanagar S., Naganandini S., Tuteja J.S., Naik S., Satish G., Divya K.T. (2015). Improving Oral Hygiene in Institutionalised Elderly by Educating Their Caretakers in Bangalore City, India: A Randomised Control Trial. Can. Geriatr. J..

[119] Nihtilä A., Tuuliainen E., Komulainen K., Autonen-Honkonen K., Nykänen I., Hartikainen S., Ahonen R., Tiihonen M., Suominen A.L. (2017). Preventive Oral Health Intervention among Old Home Care Clients. Age Ageing.

[120] McGrath C., Zhang W., Lo E.C. (2009). A review of the effectiveness of oral health promotion activities among elderly people. Gerodontology.

[121] Manchery N., Subbiah G.K., Nagappan N., Premnath P. (2020). Are oral health education for carers effective in the oral hygiene management of elderly with dementia? A systematic review. Dent. Res. J..

[122] Nakre P.D., Harikiran A.G. (2013). Effectiveness of oral health education programs: A systematic review. J. Int. Soc. Prev. Community Dent..

